# Caries of the Spine

**Published:** 1893-10-07

**Authors:** E. Luke Freer

**Affiliations:** Late Hon. Surgeon Birmingham Royal Orthopædic and Spinal Hospital. Birmingham


### EDITOR'S LETTER-BOX.

### CARIES OF THE SPINE.

Sir,—Your issue of September 30th contains a contribution on this subject as treated at the Royal Orthopædic Hospital, London. While agreeing for the most part with the principles adopted, and acknowledging that physiological rest may be insured by the instruments described, albeit the writer admits that they have the objection of being expensive (a very important matter in hospital practice), I must take exception to his remarks on other modes of treatment, as the context implies that they have been abandoned not by the hospital in question, but also by the profession generally. Speaking of the various forms of apparatus for giving support and ensuring immobility while allowing the patient to move about, he says, "The chief of these methods were the plaster of Paris jacket and Sayre's poroplastic jacket" (*sic*) (this is the first time that I have heard Professor Sayre's name mentioned as having introduced Cocking's poroplastic jacket). "*These, however, have been entirely given up, as they have been found wanting as a means of cure.*" The italics are my own. This may be so at the Royal Orthopædic, but it is misleading to infer that such is the case in orthopædic practice generally. With all modesty I think I can claim to have had more experience with Sayre's plaster cuirass than any other English surgeon, and that experience enables me to affirm that for the treatment of spondylitis the results of the jacket, when properly applied, are more uniformly satisfactory than any other method. No other means have yet been devised which can give such perfect physiological rest. The failures that I have witnessed have been due to improper application and want of appreciation of the principles involved. Some of the attempts have been ludicrous—a few turns of flannel bandage round the ribs covered with plaster constituting the so-called "jacket." The mistake Sayre made was in stating in his monograph that any surgeon could apply the cuirass, whereas I know of no operation for which the "tactus eruditus" and close attention to details are more requisite. Where, however, the disease is complicated with psoas or other abscess, Thomas's double hip-splint answers admirably, ensuring perfect rest to the whole body, and at the same time permitting access for dressings, &c. I am pleased to see that your contributor recommends massage after consolidation has been obtained. To this I would add light exercises in the supine reclining position, especially what are designated "breathers"; these are of the utmost value for restoring the atonic muscles, and despite the remarks of one of your correspondents (Mr. F. G. Bennett) as to "the admission into the profession of a lot of people who practice massage." I trust your readers will not be deterred from adopting this now generally accepted and most important remedial agent where indicated on account of the wholesale denunciation of Mr. Bennett, who has evidently not taken the trouble to "sift the wheat from the chaff" before uttering his diatribe.—Yours truly, E. Luke Freer,

Late Hon. Surgeon Birmingham Royal

Birmingham. Orthopædic and Spinal Hospital.

